# Postnatal Cytomegalovirus Infection in a Neonatal Intensive Care Unit: A Retrospective Study

**DOI:** 10.7759/cureus.100175

**Published:** 2025-12-27

**Authors:** Inês F Fernandes, Marta Figueiredo, Denise Baganho, Mónica Marçal, Madalena Lopo Tuna

**Affiliations:** 1 Pediatrics, Hospital de São Bernardo, Unidade Local de Saúde Arrábida, Setúbal, PRT; 2 Neonatology Unit, Unidade Local de Saúde de Lisboa Ocidental, Lisbon, PRT; 3 NOVA Medical School, Universidade NOVA de Lisboa, Lisbon, PRT; 4 Comprehensive Health Research Centre, NOVA Medical School, Universidade NOVA de Lisboa, Lisbon, PRT; 5 Research, Centro Clínico Académico de Lisboa, Lisbon, PRT

**Keywords:** breast milk, cytomegalovirus, infected secretion, neonatal intensive care, postnatal infection, preterm infants

## Abstract

Introduction: Postnatal cytomegalovirus (pCMV) infection is a relevant cause of morbidity in preterm infants, particularly those born extremely preterm or with very low birth weight. This study aimed to describe the clinical manifestations, management, and outcomes of neonates with pCMV infection admitted to a neonatal intensive care unit (NICU).

Methods: We conducted a retrospective case series of 12 neonates diagnosed with pCMV infection between 2013 and 2024 in a level III NICU. Congenital cytomegalovirus (CMV) infection was excluded by negative urine testing within the first two weeks of life. Clinical, laboratory, and follow-up data were extracted from medical records.

Results: Median gestational age was 25.0 weeks, and median birth weight was 699.5 grams. All infants were breastfed prior to infection, with the first positive CMV sample detected at a median of 52 days of life. Hematological abnormalities were frequent, including neutropenia in 9/11 (81.8%) and thrombocytopenia in 7/12 (58.3%). Respiratory complications were common, with bronchopulmonary dysplasia in all cases, and intraventricular hemorrhage (IVH) occurred in eight (66.7%). Antiviral therapy was administered in 9/11 (75%) patients, achieving viral clearance in 8/9 (88.9%) treated infants. Overall mortality was 25%.

Conclusion: pCMV infection in extremely preterm infants is associated with significant morbidity, frequent hematological and respiratory complications, and high mortality. Although antiviral therapy achieved viral clearance in most treated infants, long-term neurodevelopmental impairment was common. Distinguishing sequelae attributable to pCMV from those related to extreme prematurity remains challenging, underscoring the need for prospective studies to better guide management and assess long-term outcomes.

## Introduction

Cytomegalovirus (CMV) is a common cause of intrauterine and perinatal infection. Perinatal infection occurs during exposure to maternal genital secretions at delivery or through infected secretions such as breast milk, saliva, or blood products [1-3].

The majority of CMV-seropositive mothers experience reactivation of the infection during lactation, and approximately 96% shed the virus in breast milk, without clinical or laboratory evidence of infection [1]. In most cases, CMV in breast milk can be detected about two weeks after birth, with viral load peaking at four to eight weeks postpartum [2,4].

It is estimated that CMV transmission through breast milk occurs in about 58-69% of term neonates, 5.7-58.6% of preterm neonates, and approximately 38% of very preterm neonates with birth weight (BW) <1500 grams (g) or gestational age (GA) <32 weeks [1]. Detection of CMV in neonatal urine typically occurs 4-12 weeks after birth (mean 6-8 weeks)[1].

Preterm and very low birth weight (VLBW) infants are at higher risk of CMV infection via breast milk due to their immature immune system and insufficient maternal antibodies [1]. Consequently, they may present with more severe clinical manifestations such as neutropenia, thrombocytopenia, respiratory distress, hepatitis, hepatosplenomegaly, petechiae, neonatal jaundice, gastrointestinal symptoms, or sepsis-like illness [1,5]. In term infants, postnatal CMV infection (pCMV) is usually asymptomatic and does not result in sequelae or hearing deficits [1,2].

There is no consensus regarding the treatment of pCMV in preterm infants, nor on the indications or timing for initiating antiviral therapy [1,4]. When treatment is initiated, the most recent studies suggest the use of oral valganciclovir or intravenous ganciclovir, although data on their use in infants remain limited [4]. Available evidence suggests that a four to six-week course of ganciclovir/valganciclovir may improve clinical outcomes, particularly hematological abnormalities [4]. However, it remains unclear whether improvement is attributable to antiviral therapy or to the spontaneous resolution of infection coinciding with the end of treatment [5].

## Materials and methods

This retrospective case series aimed to characterize the clinical features, management strategies, and short- and long-term outcomes of neonates with pCMV admitted to a level III neonatal intensive care unit (NICU). All data were fully anonymized before analysis, and no identifiable information was collected. This study involved the retrospective analysis of fully anonymized clinical data, and thus did not need ethics committee approval according to our institutional policy. Informed consent was waived.

Study population and sample selection

All infants admitted between January 1, 2013, and December 31, 2024, were screened for eligibility. To identify eligible cases, all NICU discharge summaries issued during the study period were reviewed. A keyword-based search including “postnatal CMV infection” and “cytomegalovirus” was performed to identify patients with a recorded diagnosis of pCMV infection. Diagnosis was confirmed using quantitative real-time polymerase chain reaction (qRT-PCR), with CMV ELITe MGB® Kit on the ELITe InGenius® system (ELITechGroup SAS, Puteaux, France) in 10 patients; in two of the earliest cases, viral isolation was conducted using the shell-vial assay (rapid cell culture technique; local laboratory method, no specific commercial kit was used), the standard method available at the time.

Neonates with congenital CMV infection, defined by viral isolation in urine samples collected within the first two weeks of life, were excluded. A positive CMV test obtained after day 14 of life was considered consistent with postnatal acquisition.

Data collection

Clinical data were retrieved from electronic medical records using a standardized collection grid. Variables included demographic and perinatal characteristics, maternal history, feeding practices, virological results, hematological and biochemical parameters, major clinical complications, treatment, and outcomes at discharge and follow-up at 12 and 24 months, if available. Some laboratory variables were missing for a subset of infants. Given the small sample size, no cases were excluded due to missing data; instead, analyses were performed using available-case denominators.

Statistical analysis

Statistical analysis was descriptive. Categorical variables were expressed as frequencies and percentages. Continuous variables were reported as median, interquartile range (IQR) and range. Analyses were performed with IBM SPSS Statistics for Windows, version 25 (IBM Corp., Armonk, New Year, United States).

## Results

A total of 12 neonates with pCMV were included, with an equal sex distribution. During the study period, 688 VLBW infants were admitted to the NICU, corresponding to a pCMV rate of 1.74% in this population. Most cases (n=10; 83%) were identified between 2021 and 2024. A summary of baseline characteristics, laboratory findings, complications, treatment, and outcomes is presented in Table 1.

**Table 1:** Clinical and laboratory characteristics of neonates with postnatal cytomegalovirus infection * Viral load quantification not available for two cases. ** In two additional patients, bronchial secretions weren’t collected. † Data available for 11/12 infants (one missing); ‡ Proportion among treated infants (n=9) ALT: alanine Aminotransferase; AST: aspartate aminotransferase; CMV: cytomegalovirus; GGT: gamma-glutamyl transferase; IQR: interquartile range

Variable	Value
Perinatal characteristics	
Male sex, n (%)	6 (50.0%)
Gestational age at birth (weeks), median (IQR)	25.0 (1.7)
Birth weight (grams), median (IQR)	699.5 (198.7)
Small for gestational age, n (%)	3 (25.0%)
Fetal growth restriction, n (%)	2 (16.7%)
Cesarean delivery, n (%)	10 (83.3%)
Apgar score at 1 minute, median (IQR)	6 (2.75)
Apgar score at 5 minutes, median (IQR)	8.0 (1.75)
Apgar score at 10 minutes, median (IQR)	9.0 (1.0)
Maternal age (years), median (IQR)	33.0 (6.3)
Siblings present, n (%)	4 (33.3%)
Virological data	
First positive CMV sample (day of life), median (IQR)	52.0 (25.3)
Peak urine CMV load (IU/L), median (IQR)	749,713.5 (3,372,993.3)*
Day of peak urine load (day of life), median (IQR)	55.0 (32.0)*
Peak plasma CMV load (IU/L), median (IQR)	10,864.0 (257,622.0)*
Day of peak plasma load (day of life), median (IQR)	53.0 (23.0)*
Peak bronchial secretion CMV load (IU/L), median (IQR)	58,536.5 (463,486.0)**
Day of peak bronchial load (day of life), median (IQR)	54.0 (17.3)**
Feeding before infection	
Exclusive breast milk, n (%)	11/11 (100.0%)†
Hematological/biochemical findings	
Leukopenia, n (%)	5/11 (45.5%)†
Minimum leukocyte count (/mm³), median (IQR)	3,100.0 (1,300.0)
Neutropenia, n (%)	9/11 (81.8%)†
Minimum neutrophil count (/mm³), median (IQR)	470.0 (430.0)
Thrombocytopenia, n (%)	7 (58.3%)
Minimum platelet count (/mm³), median (IQR)	60,000.0 (24,000.0)
Elevated transaminases (AST/ALT >60 IU/L), n (%)	1/11 (9.1%)†
Hyperbilirubinemia, n (%)	1/11 (9.1%)†
GGT elevation, n (%)	0/11 (0.0%)†
Complications	
Respiratory distress syndrome, n (%)	5 (41.7%)
Pneumonitis, n (%)	4 (33.3%)
Bronchopulmonary dysplasia, n (%)	12 (100.0%)
Sepsis, n (%)	2 (16.7%)
Intraventricular hemorrhage, n (%)	8 (66.7%)
Abnormal hearing screening, n (%)	4 (33.3%)
Treatment and outcomes	
Antiviral treatment (valganciclovir), n (%)	9/11 (75.0%)†
Duration of treatment (weeks), median (IQR)	7.0 (2.1)
Plasmatic CMV negativity achieved, n (%)	8/9 (88.9%)‡
Time to CMV negativity (days), median (IQR)	28.0 (14.0)
Length of stay (days), median (IQR)	158.0 (76.8)
Mortality, n (%)	3 (25%)

Median gestational age at birth was 25.0 weeks (IQR 1.7; range 24-28) and median BW was 699.5 g (IQR 198.7; range 530-910). Three infants (25.0%) were small for gestational age, and 2 (16.7%) had fetal growth restriction. Median maternal age was 33.0 years (IQR 6.3; range 22-39). Four infants (33.3%) had siblings. Cesarean delivery occurred in 10 (83.3%). Median Apgar scores were 6.0 (IQR 2.75; range 3-8) at one minute, 8.0 (IQR 1.75; range 5-9) at five minutes, and 9.0 (IQR 1.0; range 8-9) at ten minutes.

The first positive CMV sample was detected at a median of 52 days of life (IQR 25.3; range 32-75). Median peak viral loads were 749,714 international units per liter (IU/L) in urine (IQR 3,372,993; range 2,607-26,580,398), 10,864 IU/L in plasma (IQR 257,622; range 233-3,034,686), and 58,537 IU/L in bronchial secretions (IQR 463,486; range 2,394-1,601,228), at a median of 55 days (IQR 32; range 32-122), 53 days (IQR 23; range 37-102), and 54 days (IQR 17.3; range 32-70) of life, respectively. All infants were exclusively breastfed prior to infection (feeding data were unavailable for one patient).

Laboratory data were incomplete for two patients, in whom diagnosis had been established by viral culture (shell-vial assay), the standard method at the time. Consequently, viral load quantification (urine, plasma, and bronchial secretions) was not available for these cases. In two additional patients, bronchial secretions weren’t collected.

Hematological abnormalities included leukopenia in 5/11 (45.5%), neutropenia in 9/11 (81.8%), and thrombocytopenia in 7/12 (58.3%) infants. Elevated transaminases and total hyperbilirubinemia were each observed in 1/11 (9.1%); no gamma-glutamyl transferase (GGT) elevations were recorded.

Clinical manifestations included respiratory distress in five (41.7%) patients, all requiring escalation of ventilatory support, pneumonitis in four (33.3%), and sepsis in two (16.7%). All the affected babies developed bronchopulmonary dysplasia (BPD) (n=12; 100.0%), and eight (66.7%) had intraventricular hemorrhage (IVH). No cases of necrotizing enterocolitis or chorioretinitis were observed. Abnormal universal newborn hearing screening or auditory brainstem response was documented in four (33.3%).

Antiviral therapy (valganciclovir) was administered to 9/11 (75.0%) patients for a median of 7.0 weeks (IQR 2.1, range 4-8). One infant with severe pneumonitis additionally received ganciclovir. CMV plasmatic negativity was achieved in 8/9 treated patients (88.9%) after a median of 28.0 days of therapy (IQR 14.0, range 8-56).

Median length of hospital stay was 158.0 days (IQR 76.8, range 85-254). Three infants (25.0%) died during hospitalization. At 12-month follow-up (available in seven patients), four (57.1%) showed delayed neurodevelopment; at 24 months (available in two), both had delayed neurodevelopment.

Detailed clinical and laboratory data for each individual patient are provided in Tables 2, 3. Reference range provided in Table 3 is based on the consensus of the medical literature, including Soldin et al.'s *Pediatric Reference Intervals*, 6th Edition [6], large-scale database studies, and pediatric reference interval studies [7-9].

**Table 2:** Baseline characteristics and virological data of neonates with postnatal cytomegalovirus infection (-) Missing data AGA: appropriate for gestational age; CMV: cytomegalovirus; GGT: gamma-glutamyl transferase; HM: human milk (breast milk); LGA: large for gestational age; SGA: small for gestational age

Case	1	2	3	4	5	6	7	8	9	10	11	12
Sex	Female	Male	Male	Female	Female	Male	Female	Male	Female	Female	Male	Male
Gestational Age (weeks)	25	25	24	25	28	26	34	26	25	24	24	25
Birth weight (grams)	729	831	654	530	845	910	605	741	624	676	723	588
SGA/AGA/LGA	SGA	SGA	AGA	AGA	AGA	AGA	AGA	AGA	AGA	AGA	AGA	SGA
Mode of delivery	Cesarean	Cesarean	Cesarean	Cesarean	Cesarean	Cesarean	Cesarean	Cesarean	Vaginal	Vaginal	Cesarean	Cesarean
Fetal growth restriction	No	No	No	Yes	Yes	No	No	No	No	No	No	No
Apgar (1/5/10)	5/7/9	3/6/8	8/8/9	4/9/9	8/9/9	7/8/9	7/8/9	6/8/9	5/7/8	6/9/9	3/5/8	5/8/8
Maternal age (years)	36	36	39	30	33	33	34	30	29	29	38	22
Siblings	No	No	Yes	No	No	No	Yes	No	Yes	No	Yes	No
Feeding before infection	HM	HM	HM	HM	(-)	HM	HM	HM	HM	HM	HM	HM
Day of first positive sample	73	75	53	38	60	51	43	32	36	59	46	66
Peak CMV load in urine (IU/L)	1,484,205	261,501	2,607	10,444	(-)	45,154	26,580,398	1,237,926	26,560	1,918,211	7,827,464	(-)
Day of peak urine load (day of life)	103	75	55	47	60	51	43	32	36	59	122	(-)
Peak CMV load in plasma (IU/L)	2,043	1,112	233	3,559	(-)	20,664	791,201	6,985	82,176	3,034,686	14,743	(-)
Day of peak plasma load (day of life)	102	75	53	43	(-)	55	46	(-)	37	60	51	(-)
Peak CMV load in bronchial secretions (IU/L)	(-)	(-)	178	16,293	(-)	2,394	561,484	6,510	100,780	1,601,228	183,184	(-)
Day of peak bronchial secretions load (day of life)	(-)	(-)	57	54	(-)	55	46	32	37	70	54	(-)

**Table 3:** Clinical manifestations, complications, treatment, and outcomes (-) Missing data; (*) Not applicable AHS: abnormal hearing screening; ALT: alanine aminotransferase; AST: aspartate aminotransferase; BPD: bronchopulmonary dysplasia; CMV: cytomegalovirus; GGT: gamma-glutamyl transferase; IVH: intraventricular hemorrhage; NEC: necrotizing enterocolitis; RDS: respiratory distress syndrome; ROP: retinopathy of prematurity

Case	Reference range (> 14 days of life)	1	2	3	4	5	6	7	8	9	10	11	12
Leukopenia (minimum count/mm³)	Leukocytes 5,000-19,500	No	No	Yes (3,100)	Yes (2,300)	No	No	No	Yes (3,200)	Yes (3,500)	No	Yes (1,800)	(-)
Day of minimum leukocyte count (day of life)	(*)	(*)	(*)	53	72	(*)	(*)	(*)	50	37	(*)	36	(-)
Neutropenia (minimum count/mm³)	Neutrophils 1,000-8,500	Yes (880)	Yes (920)	Yes (470)	Yes (370)	Yes (320)	No	Yes (300)	Yes (540)	Yes (600)	No	Yes (40)	(-)
Day of minimum neutrophil count (day of life)	(*)	71	87	55	37	66	(*)	68	50	55	(*)	37	(-)
Thrombocytopenia (minimum count /mm³)	Platelets 150,000-400,000	No	No	No	Yes (23,000)	Yes (69,000)	Yes (61,000)	No	Yes (41,000)	Yes (65,000)	Yes (54,000)	No	Yes (60,000)
Day of minimum platelet count (day of life)	(*)	(*)	(*)	(*)	57	66	51	(*)	47	37	61	(*)	66
Elevated transaminases (AST/ALT IU/L)	AST 16.1-55.4 / ALT 6.94-24.8	No	No	No	No	No	No	Yes (78/70)	No	No	No	No	(-)
Hyperbilirrubinemia (maximum total bilirubin mg/dL)	< 1.0	No	No	No	No	No	No	No	No	No	No	Yes (9,9)	(-)
Complications	(*)	BPD, IVH, ROP, AHS	BPD, ROP	BPD, IVH, ROP	RDS, BPD, ROP, AHS, sepsis	IVH, BPD, ROP	RDS, BPD, IVH, ROP, pneumonitis	IVH, BPD, ROP, AHS	RDS, BPD, ROP, pneumonitis	IVH, BPD, ROP	RDS, BPD, IVH, sepsis, pneumonitis, AHS	RDS, BPD, ROP	BPD, IVH, Pneumonitis
Antiviral treatment	(*)	None	None	Valganciclovir	Valganciclovir	(-)	Valganciclovir	Valganciclovir	Valganciclovir	Valganciclovir	Valganciclovir	Valganciclovir	Valgancivlovir and ganciclovir
Duration (weeks)	(*)	(*)	(*)	4	5	(-)	8	7	8	7	7	8	6.8
CMV negativity (day of life / treatment day)	(*)	(*)	(*)	(-)	Yes (62/22)	(-)	Yes (75/24)	Yes (99/56)	Yes (65/32)	Yes (69/33)	Yes (96/36)	Yes (146/21)	(-)
Length of stay (days)	(*)	253	186	151	165	85	191	117	89	91	173	131	254
Mortality	(*)	No	No	No	No	No	Yes	No	Yes	No	No	No	Yes
Follow-up 12 months	(*)	Yes (abnormal neurodevelpment	Yes	Yes	Yes	Yes (abnormal neurodevelpment)	(*)	Yes (abnormal neurodevolpment)	(*)	(-)	(-)	Yes (abnormal neurodevelpment)	(*)
Follow-up 24 months	(*)	(-)	(-)	(-)	(-)	Yes (abnormal neurodevelpment)	(*)	(-)	(*)	(-)	(-)	Yes (abnormal neurodevelpment)	(*)

## Discussion

There is growing clinical concern regarding the diagnosis of pCMV infection in preterm infants. This trend is driven by accumulating evidence that pCMV is common in this population, particularly among those exposed to breast milk from CMV-seropositive mothers, and is associated with significant morbidity. Reported rates of postnatal CMV infection in breastfed neonates vary widely, ranging from 5.7% to 60% [2]. This apparently low incidence likely reflects underrecognition of postnatal CMV infections that present minimal or nonspecific clinical signs, especially in preterm infants with coexisting morbidities.

Infection is more frequent in extremely preterm infants, with rates of 57.1% in neonates born at 23-24 weeks, 17.1% at 25-26 weeks, 15.2% at 27-28 weeks, and 7.1% at 29-30 weeks gestation [2]. Available cohort studies and reviews focus almost exclusively on breastfed populations, and none of the cited literature provides direct prevalence data for non-breastfed infants. Indirect evidence suggests that pCMV infection is rare among non-breastfed infants, as alternative transmission routes (such as blood transfusion or contact with maternal saliva) are considered minor compared to breast milk [3].

Consistent with previous studies, our cohort mainly included extremely preterm neonates, with a median gestational age of 25 weeks and a median BW of 699.5 g. These patients are particularly susceptible to severe manifestations and complications of pCMV infection due to immature immunity and insufficient maternal antibody transfer [1,2]. In our study, all infants received breast milk, and the timing of infection in our study, a median of 52 days of life, is also aligned with prior descriptions of symptomatic pCMV emerging between 49 and 58 days in infants born at 25-33 weeks [2,10].

The literature suggests that pCMV infection in preterm neonates may present with sepsis-like manifestations, including apnea, bradycardia, pneumonitis, hepatopathy, cholestasis, neutropenia, thrombocytopenia, and gastrointestinal disturbances ranging from feeding intolerance to necrotizing enterocolitis [2,10-14]. Our findings are largely consistent with these observations, although no cases of necrotizing enterocolitis or cholestasis were identified in our cohort.

Clinical presentation in our series was heterogeneous but severe in many cases. Hematological abnormalities were frequent, with neutropenia in 9/11 (81.8%) and thrombocytopenia in seven (58.3%) patients. Neutropenia appeared more common in our cohort compared with reported rates of approximately 34% [2,10]. Thrombocytopenia occurred at rates similar to the reported 60-66% [2,10]. Liver enzyme elevation and direct hyperbilirubinemia were rare, each occurring in 1/11 (9.1%) infant, which is considerably lower than the 61-66% cholestasis reported in other series [2,10-11]. These laboratory abnormalities are typically transient [2,10-11].

Respiratory deterioration around the time of diagnosis is well documented, with 15% requiring re-intubation and 28% requiring increased oxygen support [10,12,13]. Our cohort demonstrated higher severity, with respiratory distress in five (41.7%) infants, all requiring escalation of ventilatory support. The extreme prematurity in our population may have amplified the clinical expression of pCMV.

IVH occurred in eight infants (66.7%). However, prior studies report similar IVH rates between infected and uninfected infants [10,14], and no causal link between pCMV and IVH has been established. As our study is retrospective and lacks matched controls, the higher IVH rate likely reflects the extreme prematurity of the cohort rather than a direct effect of CMV.

Sepsis-like presentations occur in roughly 29% of pCMV cases [10]. In our cohort, sepsis-like symptoms attributable to CMV were identified in two infants (16.7%), which falls toward the lower end of reported ranges, likely reflecting stricter attribution criteria, excluding infants with suspected or confirmed bacterial infection in addition to pCMV infection.

These findings underscore the vulnerability of extremely preterm infants to severe clinical manifestations following pCMV infection. Recognition of these complications has heightened clinical awareness and led to more frequent consideration of pCMV in the differential diagnosis of clinical deterioration in preterm infants, particularly in those with unexplained respiratory worsening, cytopenias, or hepatic dysfunction [10]. We believe this increased diagnostic vigilance contributed to the higher number of cases identified between 2021 and 2024, reflecting both strengthened clinical suspicion and the broader use of molecular testing in our unit in recent years.

In our cohort, all patients developed BPD. Postnatal CMV infection is associated with an increased risk of BPD in preterm infants, with rates up to 90% and studies showing an adjusted odds ratio of 3.52 of BPD among CMV-positive infants compared to matched controls, even after adjusting for gestational age and BW. However, the baseline risk of BPD in infants born at 24-28 weeks and <1000 g is already very high, and the contribution of CMV infection must be interpreted in the context of these underlying vulnerabilities. While postnatal CMV infection likely contributes to the pathogenesis of BPD through direct viral cytopathic effects and increased inflammatory response, the outcome in this cohort is best explained by the combined effects of extreme prematurity, VLBW, and postnatal CMV infection, rather than CMV infection alone [12,13].

Antiviral therapy with valganciclovir was administered to nine neonates (75%), leading to CMV negativity in 8/9 treated infants after a median of 28 days. Evidence suggests virologic suppression and clinical improvement typically occur within one to two weeks of starting ganciclovir or valganciclovir in extremely preterm infants with postnatal CMV infection, but close monitoring for toxicity is essential [15]. Despite treatment, mortality was 25%, which is higher than the 1-4% mortality directly attributed to pCMV [10] but within the 6-27% range expected for extremely premature and VLBW infants [4]. This suggests that mortality in our cohort was more likely driven by extreme prematurity and comorbidities than by pCMV itself, aligning with existing literature [4,10,14].

Long-term follow-up revealed neurodevelopmental delay in the majority of assessed survivors (4/7 at 12 months; 2/2 at 24 months). These outcomes suggest that while antiviral therapy may reduce viral load, extremely preterm neonates remain at high risk for morbidity and mortality following pCMV infection, although results in the literature remain inconsistent. In a 12-year retrospective analysis of extremely premature infants (<29 weeks of gestation), rates of normal hearing, cognitive and motor development, and neurologic impairment at 1.5-2 years of corrected age did not differ between those treated with antivirals and those who were untreated or CMV-negative [4]. There is no evidence from comparative studies that antiviral treatment improves long-term neurodevelopmental outcomes, hearing, or mortality in extremely preterm infants with postnatal CMV infection, and the decision to treat should be individualized, weighing potential benefits against risks [4]. Thus, our observations expand upon existing evidence by reinforcing the persistent uncertainty regarding the independent contribution of pCMV to long-term outcomes and highlight the need for continued monitoring and further research to clarify the long-term impact of postnatal CMV infection. As in prior work, the effects of extreme prematurity and VLBW likely play a major role, complicating attribution.

Although maternal CMV serostatus and breast milk testing were not assessed in our study, all infants received breast milk, making milk-mediated transmission a plausible route. Various interventions, including freezing, pasteurization, microwave, and UV-C irradiation, can reduce CMV viral load in HM, but complete eradication is not guaranteed [2]. Nevertheless, current guidelines continue to recommend breastfeeding for both term and preterm neonates, balancing the potential risks of pCMV infection against the well-established benefits of breast milk [5]. In our unit, no specific treatment or processing of breast milk is routinely performed.

It is important to emphasize that many of the observed morbidities, particularly neurodevelopmental outcomes, may also result from extreme prematurity and VLBW. Distinguishing the specific contribution of pCMV infection from complications inherent to prematurity, therefore, remains challenging.

This study is limited by its retrospective design, which relies entirely on existing clinical records. As a result, the completeness and quality of follow-up data, including long-term neurodevelopmental outcomes, hearing, vision, and growth, are limited. The small sample size further restricts the ability to perform statistical analyses to identify risk factors or predictors of outcome. Therefore, our findings are purely descriptive. Additionally, maternal CMV serostatus and CMV testing of breast milk were not routinely performed, thereby limiting confirmation of the exact transmission route.

Overall, our findings support prior evidence of significant morbidity associated with pCMV in extremely preterm infants while also highlighting the difficulty in separating the effects of CMV from those of prematurity. Prospective studies on screening and treatment will help clarify whether these interventions offer additional benefits for preterm infants, who are already a high-risk population. Importantly, systematic testing of VLBW neonates within the first three weeks of life remains essential to exclude congenital CMV infection and to accurately differentiate congenital from postnatal disease [16].

## Conclusions

Postnatal CMV infection in extremely preterm neonates is associated with high rates of hematological abnormalities, respiratory distress, bronchopulmonary dysplasia and other complications. Antiviral therapy with valganciclovir was frequently administered and led to viral clearance in most treated infants. Mortality remains significant in this vulnerable population. Although long-term follow-up data are limited, a substantial proportion of survivors showed delayed neurodevelopment at 12-24 months. Nonetheless, these outcomes must be interpreted with caution, as distinguishing the sequelae of pCMV infection from those related to extreme prematurity and VLBW remains difficult. These findings underscore the need for careful monitoring of preterm infants exposed to CMV and highlight the importance of further studies to guide antiviral therapy and assess long-term outcomes.
